# Impact of Quality Improvement and Milling Innovations on Durum Wheat and End Products

**DOI:** 10.3390/foods11121796

**Published:** 2022-06-18

**Authors:** Ashok Sarkar, Bin Xiao Fu

**Affiliations:** 1 Cereals Canada, 1000-303 Main Street, Winnipeg, MB R3C 3G7, Canada; 2 Grain Research Laboratory, Canadian Grain Commission, 303 Main St., Winnipeg, MB R3C 3G8, Canada; binxiao.fu@grainscanada.gc.ca

**Keywords:** durum wheat, semolina milling, semolina quality, granulations, digitalization, sustainability

## Abstract

There are long-standing established intrinsic quality requirements of end products made from durum wheat semolina, with color, textural, and cooking properties of pasta and couscous representing persistent key attributes for consumers. Over time, traditional efforts to advance development in these areas with respect to raw material, equipment, and process improvements have been influenced by growing awareness of issues around food safety, health and nutrition, and climate change, necessitating that development strategies incorporate specific considerations relating to safety, traceability, and sustainability. We examined improvements in durum wheat quality and innovations in milling and the resulting impact on product quality in light of these considerations, which are now fundamental to the planning and development of any food process, as required by consumers and regulators alike.

## 1. Introduction

Durum wheat is an important crop that serves as a staple and a good source of nutrition for consumers around the world. Durum wheat is widely utilized in diverse traditional food products consumed in the Mediterranean basin [1]. Globally, durum wheat semolina is considered to be the most suitable raw material of choice for pasta, couscous, and a variety of breads due to its natural pigment color, hard kernel texture, and good protein content and quality. These properties are directly attributable to the intrinsic quality of the durum wheat that is milled into semolina. Furthermore, these end-use properties are influenced by various milling processes, and quality optimization requires an understanding of the specific effects of these processes. Other factors, including the successes of the breeding program, have also contributed to quality improvements in recent years.

In an environment of increasing awareness around issues of food safety and sustainability, desirable characteristics of semolina products now extend beyond quality and functional attributes, with increasing emphasis on food safety issues (e.g., cadmium and DON), as well methods for sustainable production. Processing equipment advances such as the use optical sorters and pearling systems have improved the elimination of undesirable and toxic extraneous materials found in incoming wheat, while the widespread adoption of fine semolina granulation and design advances relating to roller mills, sifters, and purifiers have allowed for improved energy conservation without sacrificing quality or safety. Additionally, online quality control of wheat and products and application of advanced automation through digitalization hold considerable promise toward further improving productivity, energy efficiency, process optimization, and reduced wastage.

In this paper, we examine and discuss important factors relating to the current state of durum wheat production, milling, and processing, with particular attention to advances in the areas of product quality, safety, process optimization, and sustainability.

## 2. Advances in the Quality of Durum Wheat

Durum wheat is widely used in various products, including long and short dried pasta, fresh and sheeted pasta, couscous, and baked bread. High-quality durum wheat has superior milling quality, producing a high yield of semolina with low ash content and speck count, and has high yellow pigment content necessary to produce products with a bright yellow color. A high protein content and strong gluten characteristics ensure superior pasta-cooking quality and good performance in certain products, such as durum bread. New durum varieties expressing low cadmium uptake are required to meet the food safety regulations in many markets.

For high-quality production of semolina, the selection of appropriate quality of the raw material—durum wheat—is vitally important. Recent advances in the availability of improved durum wheat varieties have been a great success story in raising the standard of quality, which is higher than ever before, particularly with respect to the following key quality attributes.

### 2.1. Protein Content

Durum wheat with a high protein and good physical condition will generally yield semolina of uniform particle size with a minimum number of starchy particles [2]. Protein in semolina facilitates hydration during mixing and provides the structure for pasta. A high protein concentration is the prerequisite for superior pasta-cooking quality. The protein content is a major determinant of the value for durum milling and pasta processing [3].

Both genetic factors and environmental conditions influence the protein concentration in durum wheat. Growing varieties with a high protein potential is an effective way to maintain grain protein content at the highest possible level in a low-input production system characterized by low rates of nitrogen fertilizer application [4].

### 2.2. Gluten Strength

Gluten strength has been widely considered as an important secondary prerequisite for superior pasta-cooking quality [5]. The continuity and strength of the protein matrix formed during extrusion are important in determining the textural characteristics of the pasta. The relationship between gluten strength and pasta-cooking quality is complex and inconclusive. Furthermore, there is strong evidence that, under high-temperature drying conditions, gluten strength has less influence on the pasta’s cooking quality than under low-temperature drying conditions [6,7]. High-temperature drying is predominant in today’s pasta industry.

Regardless of whether gluten strength might be overestimated as a cooking quality prerequisite, particularly for pasta dried at a high temperature, there has been increasing emphasis placed on strength as a durum wheat quality specification. Efforts in improving durum wheat gluten quality have resulted in a significant increase in the gluten strength in the last 20 years (Table 1). It is important to have strong and extensible gluten to be suitable for most products, including long and short goods, fresh pasta, sheeted pasta, couscous, baked bread, etc. [8]. Durum wheat with inextensible gluten has limited applications. They can cause processing problems (e.g., sheeted pasta) or result in poor product quality (e.g., bread).

### 2.3. Pigment Content and Pigment Loss

One of the most important quality factors in durum wheat is the potential of producing semolina and pasta products with a bright yellow color. Semolina and pasta yellowness is affected by various factors: the yellow pigment content of the grain; the oxidative degradation of pigments by lipoxygenase (LOX) during pasta processing; and the processing conditions such as drying temperature, extrusion die design, and type [9,10].

Over the past few decades, efforts to improve the yellow color of durum semolina in durum breeding programs have resulted in the release of cultivars with high pigment levels, as reflected by the Canadian durum export cargo quality monitoring (Table 1). Market feedback has been very positive for this improvement. There is usually a slight elevation of pasta redness with the increase of total yellow pigments in durum wheat. However, the increase in pasta yellowness more than compensated for the elevation in redness in the overall appearance of pasta. Millers also use high pigment durum for blending with wheat of low pigment to improve the color of semolina and pasta products.

Some of the pigments in semolina will be degraded and lose yellow color during pasta processing through oxidation induced by LOX. Significant progress has been made in the genetics of LOX to facilitate durum wheat improvement by developing new cultivars with low LOX activity [11]. The allelic variation for a deletion of the *Lpx-B1.1* was associated with a significant reduction in LOX activity and improved pasta color due to reduced pigment degradation during pasta processing [12]. While high pigment content is the primary factor for superior color of semolina and pasta, a reduction in LOX activity will further improve the color of pasta products.

### 2.4. Milling Performance

Milling performance is the most important factor that determines the industrial value of durum wheat. The key indicators of milling quality are yields (total and semolina), ash content, and speck counts in the finished granular product. Yield is a key indicator of profit for durum mill. There is a legal limit for semolina ash content in some EU countries. The speck count is a deciding factor of consumer acceptance for many durum products. Durum wheat with superior milling quality is characterized by high test weight, large kernels, and high percentage of hard vitreous kernel (HVK). Table 2 listed tolerances of some key grading factors for Canadian durum wheat.

The physical defects associated with surface discoloration of kernels are important because bright speck-free semolina is required to give the aesthetic appearance of premium semolina and pasta products. Kernels with surface discoloration are tolerated in very low amounts in high-quality durum wheat. Ergot sclerotia, smudge, black point, mildew, and midge are the main physical defects associated with surface discoloration [13]. Resistance to disease and insect damage not only prevents loss of yield but also protects the grade and quality of the grain, especially for durum wheat, because of the major impact on speck count.

To ensure that Canada Western Amber Durum (CWAD) wheat meets the quality and safety expectation of customers, stringent tolerances are set for various milling grades.

### 2.5. Cadmium Level

High levels of Cd in cereal grains are a health concern. Durum wheat normally accumulates more Cd than other commonly grown cereals, with concentrations ranging from less than 30 to more than 300 μg/kg [14]. A low Cd concentration in durum wheat is mainly controlled by a single dominant gene (Cdu-B1) that is highly heritable [15]. Incorporation of the low Cd allele into cultivars reduces the grain Cd by about 50%. Low Cd is mandatory for registration of durum wheat cultivars in Canada [3]. The environment and soil conditions, however, play an important role in Cd content in the grain, even for low-Cd varieties.

The Codex General Standard for Contaminants and Toxins in Food (Rev. 5, 2009) lists the maximum limit for Cd in wheat grain as 200 ppb. EU has instituted a new standard for Cd at 180 ppb for durum wheat since August 2021 [16]. The current guide of <100 ppb for registering new durum varieties in Canada can effectively manage the Cd level.

### 2.6. Future Trends in Durum Quality Improvement

Fusarium head blight (FHB) is a major fungal disease. Durum is notorious for its extreme susceptibility to FHB and breeding for FHB resistance is difficult. FHB has a great economic impact on durum crops due to the reduced seed quality and agnomical yield. Semolina yield, gluten strength, and pasta quality can adversely be affected by the presence of fusarium damaged kernels (FDKs), which are usually shriveled [13]. The current FDK tolerances for grading Canadian wheat (Table 2) can effectively ensure durum wheat milling performance and semolina and pasta quality. FDK is also highly problematic because of fusarium mycotoxins, which render the grain unfit for food and feed. Genetic resistance is the most cost-effective and environment friendly approach for controlling FHB and development of new cultivars with improved resistance is the major goal for improving durum wheat quality and safety [17].

Drought and heat are major abiotic stresses affecting durum wheat production and quality worldwide. In addition to the yield loss, the effects of drought and heat stresses have a very significant impact on durum quality, as indicated by a lower test weight, smaller kernels, lower milling yield, and higher semolina ash [18]. Enhanced heat and drought tolerance of wheat is not only to ensure a stable yield across both good and bad seasons while maintaining a high yield under optimal conditions, but also to protect quality by minimizing their impacts on kernel morphological properties, which, in turn, adversely affect milling quality.

Protein content is critical for kernel virtuosity and pasta-cooking quality [2]. Nitrogen fertilizer is the most used nutrient source in modern agriculture and represents significant environmental and production costs. The selection of new varieties with higher nitrogen-use efficiency has become of ever-increasing importance.

## 3. Raw Material and Semolina Milling

Semolina milling operations is a vital link between durum wheat and quality end products such as pasta, couscous, and popular Mediterranean baked goods. While semolina quality is strongly correlated to the intrinsic quality of the raw material—wheat—the processing environment plays a major role in determining the outcome of the final quality of semolina consistently over the long production periods.

Starting with high-quality raw material characterized by a good kernel size, high test weight, and high percentage of hard vitreous kernels (HVKs), along with all the appropriate physical and functional quality attributes, is the best approach to produce good quality semolina. The value of good and uniform kernel size has been emphasized in the literature for good milling performance [19]. The relationship of milling properties with physical properties of wheat kernel has been studied extensively. Djiki and Laskowski (2005) [20] stated that the 1000 kernel weight in durum wheat is associated with semolina yield and test weight. They also suggested that kernel size uniformity is very important in the milling process with respect to cleaning, conditioning, debranning, and grinding. Recent research on the relationship of kernel size and genotype on milling performance further substantiates the importance of these aspects for the selection of appropriate durum wheat [18]. Often the semolina quality specified requires the blending of two or more wheat types to achieve the desired quality of the blend. The motivation for blending may also be to reduce the cost of wheat mix. This may also introduce the differences in kernel sizes if the wheats in the blend are of variable sizes. Major durum-wheat-exporting countries maintain their quality standard by implementing a numerical grading system, with tolerance levels set for reflecting quality, as previously shown in Table 2.

The next critical step is in the milling process, which allows millers to generate desirable levels of semolina yields of specified quality. Developments in the processing of semolina has been influenced by the continued increased demand for finer semolina over the years. The stringent semolina-processing requirements of traditional coarse semolina (<630 µm) to more easily attainable finer semolina (<355 µm) have led to relative simplification of what tended to be extensive processing practices. Finer semolina granulations with wider acceptance over time have somewhat reasonable levels of processing system with improved yields. Such plants are also easier from operation and supervision point of view. There are still some markets where the demand for coarse semolina exists, along with fine semolina, and in such situations, a portion of the coarse semolina is separately ground into a finer semolina particle size, as specified [21].

The expectations of the milling-process outcome are in the production of semolina quality of required specifications consistently. The quality expectations may often be challenged for unavailability of appropriate durum wheat due to crop failure, environmental factors, logistics, or even trade disruptions.

Recent advances in the milling equipment and in the process solutions have greatly improved the situation mitigating the quality shortcoming of wheat by compensating with improved milling technology to a reasonable extent.

## 4. Wheat Preparation

The primary functions in wheat preparation include the following:

- Removal of all non-wheat material, including damaged and diseased wheat and thin, immature, shrunken, and broken kernels;
- Bringing the wheat to its optimum condition for milling with appropriate tempering or conditioning;
- Removal of surface contaminants, crease-dirt, and the loosened outer layer of the bran following conditioning.

The equipment and process involved in this area have been extensively covered by Bizzarri and Morelli (1988) [21], Kuentzli (2001) [22], Sarkar (2003) [23], and Posner and Hibbs (2005) [24]. Although there have been ongoing continued improvement and development, the basic working principles and functions have remained essentially the same. On the other hand, there have been some noteworthy major developments that have benefitted the semolina milling operations significantly and therefore deserve appropriate discussion.

Ensuring durum wheat free from all foreign materials, damage, and diseased kernels, as well as all types of defects before the milling process begins, has always been the top priority for semolina millers. This operation always received necessary attention due to its importance on performance and impact on quality. Millers have always been able to perform this function very well, although with some degree of challenges primarily related to unavailability of cleaning equipment of high-performance as available today. Essentially, what this means is that, in the “Traditional” cleaning plants, the number of equipment used for cleaning was higher than today, adding to the space requirements and energy costs. This transformation is shown in Table 3. Although it is hard to capture all aspects of transformation without being too comprehensive, the table depicts the essential elements of the major developments.

As we review Table 3, we observe that the sequence of removal of the impurities remains the same through both phases of development—“Traditional” and “Contemporary”. The principal differences are in the compactness and comprehensiveness of each new cleaning machine performing multiple functions with a high degree of efficiency and cost savings in terms of building-space and energy requirements. This is seen by observing the subsequent columns of “Contemporary” that appears to be quite lean compared to the “Traditional” plants. The “Contemporary” model is simpler and more powerful in delivering high level of performance.

The “Traditional” cleaning plants are described and discussed at length by Bizzarri and Morelli (1988) [21], Kuentzli (2001) [22], and Sarkar (2003) [23]. Some of the latest developments and innovations that are helping enormously and being embraced by the industry widely are discussed here.

### 4.1. Developments and Innovations in Wheat Preparation

Among many developments introduced by equipment suppliers, some of the noteworthy ones that deserve mentioning are Vitaris, Optical Sorters, and Pearlers.

### 4.2. Vitaris (Cleaning of Bulk of the Foreign Material)

The four functions shown in Table 3 required individual machines to perform in the older plants. The Combi-Cleaner, which was introduced in the 1990s, was able to perform all of those functions, saving space and energy [23,24,25]. Equipment manufacturers have been concentrating on improvement in energy, space, and efficiency as the basis of development of new equipment. These three aspects together help in raising sustainability to higher standards. The introduction of a multifunctional cleaning equipment “Vitaris”, available today meets those aspects of functional attributes as in Combi-Cleaner, with an added practical feature of incorporating modularity [26]. Modularity provides flexibility in terms of combining these functions in any combination of choice of the milling company. The discretion of investing in a multifunctional compact cleaning machine during the startup or adding a function at a time as the need evolves is of practical value. The newer machine, apart from offering flexibility, comes with advanced features of energy saving, space, and superior efficiency.

Such a machine removes the bulk of the foreign materials and impurities. However, there are defects and damaged wheat kernels that can only be removed by optical sorters.

### 4.3. Optical Sorters

Traditionally, the cleaning of durum wheat has been more involved and challenging compared to common wheat cleaning [25]. The development of optical sorters has simplified the cleaning operation, while improving the cleaning efficiency enormously. The visual quality of durum wheat semolina is of the utmost importance for its end users. A bright yellow-colored semolina with minimal discoloring specks is considered to be an integral part of quality. Any black and brown specks would be conspicuous and can be seen as indications of inferior, contaminated, and impure product. With coarser granulation of durum semolina, the potential size of such specks is going to be large, as well. The presence of these specks can be minimized by thoroughly cleaning the wheat free of all types of impurities and foreign materials. Dark seeds, along with removal of wheat kernels with surface discoloration as in blackpoint and smudge, must be removed, because, apart from creating dark specks, they also impact the pasta color (Dexter and D’Egidio 2012) [27]. A durum-wheat-cleaning facility of about 15 years or more (pre-optical sorter in wheat cleaning) could not remove discolored wheat kernels from clean wheat stream, as they could not be removed by any means since they are part of the wheat kernels. The only way to control this problem was to work with top grades of durum wheat that would restrict the presence of such undesirable defects as part of the grade standards. Another problem was the effective removal of oats, barley, and ergot [23] from durum wheat kernels, as their length would be quite comparable. The only difference was that they would be slightly lighter in density relative to durum wheat. This problem was overcome through the installation of more gravity-based cleaning equipment, such as gravity tables and table separators, making the durum cleaning operation more complex than seen in common wheat. Typically, investment in the cleaning house of a durum mill has been higher compared to that for common wheat for flour production.

The optical sorters helped in the removal of these impurities (ergot, oats, and barley), thereby reducing the number of gravity-based cleaning machines, while minimizing the creation of dark specks. This aspect of quality (black and brown specks) is controlled by the implementation of speck count specifications used for benchmarking semolina quality [28]. The number of black and brown specks limits in semolina required by end users varies amongst companies and from region to region, based on specific requirements of the company, grades of semolina, and method used for its determination [24]. This demonstrates the importance of aesthetics in semolina, as it directly impacts the visual appearance of pasta and couscous products. These products, including the semolina (for home use), are sold in transparent packages or packages with windows, and any discolored specks show up clearly, thus rendering the product unattractive at the point of sale.

With the application of an optical sorter, the problem of dark specks has been managed well, along with replacements of the mechanical equipment of disc separators and indented cylinders and at a reduced energy cost [29]. There are a number of manufacturers of optical sorters in the industry, namely Bühler and Satake, among others.

Apart from assisting in quality improvements, optical sorters are very important from a food-safety point of view. There is growing consumer awareness about allergens and demand for product safety. Whole-grain semolina is more susceptible, as there is no further refinement in the milling process for removal of pieces of any allergenic material that goes into the milling process, along with durum wheat.

The following contaminants that are considered a risk to human health are effectively reduced by optical sorters, making it an important machine from a food-safety point of view.

- Allergens such as peanuts, soy splits, and pieces of soy;
- Mycotoxins such as vomitoxin (DON) through the removal of fusarium-damaged kernels [29];
- Ergot bodies;
- Wide range of foreign materials.

Soy pieces or splits, along with whole soy, apart from being allergens, can also result in the light bleaching of pasta dough made from durum semolina during the mixing stage in a slow and longer mixing environment. The bleaching could become extensive in the case of semolina and flour when being mixed for bread dough. This is due to lipoxygenase activity, which is undesirable for the end user.

The effective removal of ergot [30] reduces the generation of dark specks, and, more important, the removal of ergot bodies eliminates the potent alkaloids associated with them that are a risk to human health [27]. The improved detection of subtle color differences is also very helpful in removing fusarium-damaged kernels from durum wheat, helping reduce the mycotoxin levels of deoxynivalenol (DON) [30]. Fowler described the advancements in optical sorters, from monochromatic to bichromatic application enhancing the detection of subtle color differences. Fowler further stated that innovations in optical sorting have effectively removed fusarium-affected wheat from good-quality wheat.

The recent advances in optical sorters have been noteworthy. Technological developments have resulted in the improvement of performance, reliability, flexibility, ease of use, and connectivity. The new optical sorters by Sortex [31] feature improved hardware and software, along with advanced sorting algorithms that help to optimize machine performance. Developments involve improvements in the design of a camera with a low signal-to-noise ratio. The optical sorter aided by full-color cameras with enhanced spectral purity and higher-intensity LED lights helps improve defect detection. Multilayered algorithms, along with precision ejectors, reduce losses of good wheat kernels, with the rejects resulting in higher yields. All the improvements, as mentioned above, have helped improve defect detection quality while reducing losses of good wheat kernels, along with the rejects. Wheat cost is the largest single cost in the production of semolina, saving even a small quantity of wheat would result in substantial savings. Such a precise sorting capability helps in the utilization of lower-quality wheat as input with acceptable output quality.

Connectivity facilitates the monitoring and control of the machine from anywhere, ensuring high performance and product traceability, along with host of other benefits that include improved productivity, quality, downtime, and reduction of operation costs.

### 4.4. Pearling

#### Surface Treatment of Durum Wheat

The last step in the preparation of durum wheat for milling involves scouring or surface treatment. This has been traditionally an important step in the preparation of durum wheat for milling as pasta processors require a low microbiological count for semolina. Typically, intensive scouring was used for this purpose until debranners or pearling systems were found to be more beneficial and effective for the following reasons:

- Removal of significant percentage of bran layers of ~8% in one step, thereby removing surface dirt and contaminants and reducing microbial counts;
- Removal of bran layers help reduce bran content to deal with during milling;
- Significantly increasing the semolina yield [32];
- Improving the quality of semolina in terms of low speck count and ash content as dark spots are removed with the bran layers;
- Improvements in pasta brightness and color [32].

Considerable work in the application of the debranning process, developed by Satake [33], was carried out, and its beneficial impact on durum wheat processing was reported [32,34]. Various equipment-manufacturing companies have developed such equipment of their own based on a similar concept.

This process has become a standard feature now in most durum semolina mills around the world. The durum wheat kernels with a larger size and hard kernel texture lend themselves to be easily pearled than common wheat.

The early generation of the pearling system steadily gained popularity amongst the durum millers due to their improved performance. One such system was described by Gruber and Sarkar (2012) [25]. A newer version of this machine (OSIRIS) offers further improvements with more durable, low-energy, and more effective pearlers by replacing stone grinding with diamond-coated grinding wheels, reducing the contamination of stone particles due to wear. This upgrade improved the pearling degree with minimal breakage, while enhancing the product quality and food safety to a very high standard [31,35].

Pearling/debranning has been researched extensively apart from its central theme of improving milling performance, semolina yield, and quality. The benefits of pearling have been further investigated in the reduction of alpha amylase in wheat and its products following pearling [36,37]. The effects of industrial processing on the distributions of deoxynivalenol, cadmium, and lead in durum wheat milling fractions were also investigated by Cheli, 2010 [38]. Cheli et al. concluded that there was more of a reduction of contamination in milled fractions destined for human use from debranned wheat as compared to wheat milled without debranning. They also proposed that debranning wheat would be even more relevant when working with raw materials with a contamination level closer to the legislated levels. It was noted that the pericarp and testa together, being the peripheral part of the grain, are first colonized by the fungi and often contaminated by the microorganisms, heavy metals, and soil. The debranning/pearling process, in addition to improving semolina yield and quality, also helps in reducing any such contamination present in durum wheat.

As noted earlier, with about 8% of bran being removed in the pearler (debranner), there is less bran to deal with in the mill. This allows the break-system to be simplified: since most of the coarse bran is removed, there is less need of coarse break grinding passages [24,25]. For example, instead of fourth break passage coarse (4BC) and fine (4BF), there may be just one fourth break and, likewise, one fifth break instead of fifth break coarse and fine grinding passages. There may also be fewer sizing passages. Generally, similar milling surfaces remain, as the diagram does not change much. Purification passages also remain similar. The main benefit is in higher semolina yields and improved products.

The optical sorter and the pearling system together have been extremely effective in helping improve the visual quality of the semolina. The visual discoloration on account of seed contamination and defects can be reduced to a level with the help of this combination where no dark specks are visible.

## 5. Advancements in the Milling Process

Good milling performance is a function of the milling process and milling equipment. Landi (1995) [28] stated that the milling process does not improve or add to the inherent qualities of a wheat; however, it can destroy wheat quality if carried out incorrectly.

The collective effort of selection of good-quality wheat, its preparation for milling, and superior milling performance is vital for product quality, output, and semolina yield.

The milling process is key to ensuring that the following properties are maintained in the semolina produced:

- Low black and brown speck count;
- Appropriate particle size distribution (granulation);
- Low ash content;
- Low Starch damage.

### 5.1. Black and Brown Speck Count

The sources of specks, especially dark specks, are attributable to foreign material and discolored durum wheat kernels on account of heat damage with black-tipped germ, blackpoint, and smudge [24,25]. A good cleaning plant with an optical sorter and a debranner/pearler can minimize the presence of such specks, as explained in the earlier section under cleaning.

Apart from dark/black specks, brown specks are also evaluated carefully to ensure that the visual quality of semolina meets the requirements of the processor. Brown specks are created due to premature shredding of bran during milling and/or incorrect setting of machines, such as purifiers. This is coupled with the fact that coarse semolina generation would require lighter grinding, elaborate grading and purification, and a comprehensive sizing system to extract a large amount of semolina with minimal production of flour. Even then the semolina yield of good quality is in a range of around 66–68%.

### 5.2. Semolina Particle Size Distribution (Granulation)

The production of high-quality speck-free yellow color coarse semolina posed a major challenge to durum millers to meet the quality requirement [22]. As mentioned above, despite the employing elaborate break system, grading, purification, and sizing system, the semolina yield remains very limited [24,25].

The traditional semolina granulation from 35 to 40 years ago typically consisted of coarse particles with a very restricted percentage of flour (<2%) [22,25]. Traditional pasta processors showed a preference for coarse particle size with minimal flour. It has been well documented that coarse particles take longer to hydrate, and if they are not fully hydrated, this results in white spots [24,25,28]. Pasta processors’ requirements of a very low percentage of flour in the coarse semolina was likely to control the differences in granulation. Kuenzli suggested that the tight tolerance was helpful in preventing any potential adulteration of durum flour with common wheat flour, as, around that time, there were no accurate tests available to determine that [22].

Coarse semolina was also preferred in North Africa, a major durum-consuming region, for the production of couscous. The production of couscous 30 years ago was primarily carried out by hand, requiring the use of coarse semolina to facilitate the easier production of large granules of couscous (1300–500 µm) [19].

Coarse semolina particle size requires a gradual processing system to ensure that a large particle size is maintained while detaching the adhering bran particles from the large chunks of endosperm in the break system. This is followed by a long-extended grading and purification system for the removal of these detached bran pieces. Following the removal of the large pieces of semolina in the purifier, there is a good portion of material with bran pieces attached to the endosperm that still requires detaching. This is sent to the sizing system for further size reduction, carefully keeping the particle size as large as possible while detaching the bran pieces. This is followed by comprehensive purification in sizing purifiers. This entire process can be elaborate in order to maintain a larger semolina particle size; therefore, when a coarser semolina particle is required, the processing system must be more gradual in order to ensure that a large particle size is achieved. This means there is more of a need for equipment to enable the gradual processing. Vertical integration in the pasta industry has been common practice (Industry & Trade Summary 2003) [39]. As suggested by Kuenzli (2001) [22], those durum milling facilities that are part of the pasta processing plants have been benefitting from the production of fine semolina granulation containing a portion of flour. From the literature, it appears that Manser (1985) [40] presented the benefits of finer semolina granulation from the point of view of pasta processors [22,24]. Finer granulation helped with quick and uniform hydration, due to a narrow range of particle size. The emergence of a fast mixing process worked very well with finer granulations of semolina, reducing mixing time, improving the homogeneity of the extruded dough [41], and resulting in pasta products with improved color [22].

Reduced dough-mixing time and high-temperature drying works well with finer semolina granulation tripling the pasta production [28], while helping durum millers to work with simplified fine semolina production diagram [25]. Table 4 below shows the grinding passages of a mill using coarse semolina production as compared to a plant that uses pearlers and production of finer semolina. The number of corresponding passages for finer semolina over coarse semolina in each processing passages is lower, except for the “conversion”, where the finer material gets ground into flour.

Even though the vast majority of durum semolina is being produced with finer granulations, there are markets that still use coarse semolina of 630–200 µm for traditional pasta in parts of Europe. There is still a portion of coarse semolina of 1000–600 µm produced by plants in North Africa for handmade couscous.

Table 5 shows milling surface allocation for durum semolina mills with fine granulation in comparison with traditional coarse granulation. There are appreciable differences between the two practices with respect to roll and purification surfaces. Part of the reason for this may be rooted in the fact that the traditional coarse semolina was being generated in the older, less efficient equipment. It is, however, mainly due to more elaborate grading, purification, and sizing system, which are all required to achieve the desirable quality of the coarse semolina.

Durum semolina mills in North America commonly produce semolina of granulation <425 µm, which is coarser than the fine semolina granulation produced, as well <355 µm. If milling surface allocations were to be compared between these two granulations, they would be similar. For example, instead of six sizing passages, there may be just one or two passages used for fine semolina, and in place of two reduction passages, there may be four or five passages for fine semolina, as there is more flour produced. Purification passages remain similar. While dry pasta production works well with finer semolina, there is a preference for coarse semolina for fresh pasta products such as ravioli and tortellini.

The most recent developments in durum wheat milling primarily have been in the increased utilization of durum flour (<180 µm) for end products. This helps the durum miller further increase the yield to up to 78% or higher, as compared to 72% to 74% of fine semolina with some flour. There is a growing demand for the increased utilization of durum flour (<180 µm), either alone or in combination with fine semolina. It is common to see a good number of pasta packages sold in North America showing durum flour as the second major item on the ingredient list. This is paving the way in favor of increased durum flour production. Durum flour is also used for the production of artisan breads and hearth breads. Increased yield has certainly helped the profitability for the durum milling business, as a higher yield of 78% plus is very attractive compared to the lower yield of semolina of 72% to 74% and especially when compared to the traditional yields of coarse semolina of 68% that was previously obtained around 30 to 35 years ago. The value of yield is more critical in durum milling, as the return on mill-feed (by-products) both from durum wheat and common wheat is the same, while durum wheat generally tends to be more expensive.

### 5.3. Ash Content

Ash is mineral-matter residue that is left behind upon the incineration of a sample in a muffle furnace. Ash content progressively increases from the center of the wheat kernel to the bran layers. Since ash is much higher in bran than in pure endosperm, it is a good indicator of the level of refinement and therefore is often used as a measure of milling performance. A mill providing a higher semolina yield at a given ash level is considered as superior in performance over another plant that yields lower semolina at the same ash content but milling wheat from the same source. More efficient milling produces lower ash [42]. A higher semolina yield has associated elevated levels of ash content, as the endosperm is extracted from progressively closer to the bran [43]. Usually, higher extraction also results in an increased speck count; however, with good milling practice, an increase in specks can be controlled to some extent. Studies have shown higher semolina ash content affecting pasta color negatively [44,45]. Although protein content increases with higher ash content due to an increase in semolina yield, semolina quality starts to go down due to poorer color, increased speck count, and finer particle size with potentially increased starch damage levels affecting functional properties. In their study, Joubert M. et al. (2018) [45] concluded that an increase in outer layers in semolina, with increased ash content, reduced pasta brightness and yellowness due to increased brown spots and likely enzymatic activities to pasta, while the increase in arabinoxylans, as a source of reducing sugars after extrusion, led to higher red index possibly due to Maillard reaction. Therefore, they suggested optimizing pasta color by reducing the inclusion of the grain outer layers.

Baking with semolina with increased yields may result in higher water absorption and inferior dough-handling properties on account of trying to extract the remaining endosperm from bran layers. Due to its importance, there are countries that have legislated the maximum allowable ash content, as shown in the Table 6:

The ash content in wheat is variable among varieties and is also influenced by environmental factors [43]. The legal limits allow the semolina yield advantage for wheat with a low ash content.

### 5.4. Starch Damage

Due to the kernel hardness in durum wheat, starch damage is easily caused if grinding during milling is not carefully carried out. One of the reasons for coarse semolina particles produced in the traditional semolina is to avoid the risk of inflicting physical starch damage during milling. A coarse semolina particle size, on the other hand, might be difficult to fully hydrate [49]. Starch damage is an intrinsic parameter affecting dough/pasta quality during dough mixing and kneading [49]. Higher starch damage results in surface stickiness and cooking loss under low-temperature drying.

Desirable starch-damage levels can be achieved through several measures; some of them include appropriate tempering to ensure that durum wheat is not excessively hard as it enters the grinding process. Grinding pressure needs to be gradual, as too much compression of grinding rolls generates heat, resulting in a higher starch-damage level. Roll corrugations are dispositioned to cut rather than compress. Smooth rolls should not be used for semolina size reduction. The humidity level in the milling environment should not be too low. Overall, the processing should be gradual.

## 6. Advances in the Milling Equipment

The three principal pieces of milling equipment—the roller mill, plansifter, and purifier—collectively have gone through major enhancements with respect to performance, low energy consumption, minimal maintenance requirements, and greatly improved design for hygiene and sanitary standards. The focal point of design is easy accessibility of the equipment, allowing cleaning and complete emptying out of any residual material preserving superior level of sanitary standards. Other measures include insulation of walls and doors of sifter compartments for the prevention of condensation that could promote mold development. An important aspect of design is to ensure all the surfaces that the product encounters are made of stainless steel or food-grade material for avoiding contamination.

Although these improvements added functional advantages, the essential principles of operation and function very much remained the same. Two noteworthy developments that significantly contributed to enhancing technology of grinding and became commercially successful are the eight-roller mill and automated roll gap adjustment system. Automated roll-gap adjustment served as an important tool in remote operation, enabling roll gap adjustments while switching wheat mixes. The eight-roller mill, on the other hand, opened opportunities of constructing compact mills with lower investments, increasing the capacity of an existing mill retrofitting under space restrictions and lowering energy costs. Both innovations became available commercially around 1990. Fistes and Rakic (2014) [50] noted numerous advantages of using the eight-roller mill over conventional mills with respect to investment, operating, and maintenance costs. Their study demonstrated that the flour yield and ash content improved by making an appropriate increase in the aperture of the sieve. In a previous study, Fistes et al. (2008) [51] investigated the use of the eight-roller mill on the head reduction passages of a mill. With appropriate adjustment in the roll gap and sieving conditions they obtained similar results as in a conventional process, with investment costs and energy requirements being much lower in favor of the eight-roller mill. The eight-roller mill may be used in durum mills for first and second break grinding passages and for the reduction of semolina [25] or for the regrinding of semolina [22].

All three pieces of equipment are being continually improved for food safety, energy efficiency, and improved performance. As it would be a tedious task to cover most of the improvements in a table, Table 7 offers a quick snapshot of some of these improvements and the related advantages.

The advantages reported in Table 7 have food safety and sustainability efforts as a common element for all three pieces of equipment.

The roller mills of today are equipped with modern sensor technology that enables users to measure the grinding force, which, along with the flow-rate data, ensures that the grinding performance remains stable throughout ensuring the production of a consistent high-quality end product [52]. Dübendorrfer [52] stated that semolina for pasta requires consistently low starch damage to arrive at a desired dough consistency (viscosity) requiring less water absorption for energy saving during drying, as there is less water to be removed. Consistent particle size distribution at the lowest temperature would help achieve the consistent low starch damage required. With the help of built-in grinding-force sensor and temperature-monitoring option, the grinding-gap status and temperature distribution along the rolls are available.

## 7. Developments in Quality Measurements

The routine quality testing of durum wheat is carried out to ensure that the quality of incoming raw materials is within the expected range for generating the semolina quality desired. Quality testing on semolina and flour is carried out to ensure that the milling process is well adjusted to generate semolina and flour within the specifications of customers’ needs. The frequency of testing samples is considered to be a gauge of the consistency of quality. There needs to be a balance between the frequency of quality testing limited to the lab’s capacity and what can be considered as practical.

A study carried out by Cecchini et al. (2020) [53] demonstrated the advantage of a low-cost pocket-size sensor providing a short wavelength NIR range for easier measurements at the sample source over laboratory-based instruments and other expensive portable devices. Such options are good for enabling quality testing at inconveniently located sample source and helping in reducing the laboratory workload.

However, the assurance of consistency in quality can only be truly achieved when the quality is being monitored online, along with the production, as durum milling operation is a 24/7 operation. Davies and Grant (1987) [54], in a review of near infra-red analysis of food, reported the online application of NIR analysis being in the process of development and that this is expected to be one of the most important applications for the future utilization of NIR in the food industry.

The true consistency in the finished products can only be achieved through interventions as and when required, based on faults detected through alarms or notifications. This can only be achieved with the application of online monitoring real-time with the appropriate control systems activating alarm or notification in case of “out of specification” scenarios. Furthermore, automated machine settings drive the process toward the optimized targets as real-time quality-monitoring facilitates enabling of “Automatic control loops” to achieve that (Bühler Inc., Reference [31]).

The key quality attributes measured online in durum wheat, semolina, and flour are shown in Table 8.

These properties along with Falling number and Gluten index are commonly tested and reported in the laboratory of the plant. The quality of semolina is much more tolerant to low falling number in durum wheat especially for the application in pasta products [55]. This eliminates the need for its testing less of a priority in online monitoring.

### 7.1. Quality Monitoring Online

#### 7.1.1. Particle Size Distribution

One of the key quality parameters of durum semolina is the appropriate particle-size range for the desired semolina type consistently whether measuring traditional or fine semolina. Modern technology has enabled the online monitoring of this key attribute.

This system, which was introduced by Bühler Inc. [31], covers the measurement of particle size, ranging from as low as 10 µm to as high as 5000 µm. This range is wide enough to cover the requirements of all types of semolina for application in pasta, couscous, and bakery products (Table 9). There are other suppliers of similar products, but this system is advanced in terms of its customization for application in the durum-semolina process control. It applies laser diffraction and image-processing technology in combination to achieve the determination of particle size distribution continuously in the ongoing process. Any deviation to the particle size is detectable by the operating software serving as the basis for a monitored and traceable product quality.

This system has the ability for connecting with an appropriate roller mill, where the grinding gap gets adjusted upon the detection of any deviation between measured values and targeted values.

#### 7.1.2. Multi Online Analyzer Using NIR and Camera

This system is referred to as NIR Multi Online Analyzer [31]. The unit provides assurance of consistent quality, documentation, and traceability through real-time quality monitoring and recording.

The unit measures moisture, protein, and ash in wheat, semolina and flour using NIR probe. There are up to six measurement points that can be connected to one NIR spectrometer.

The modular system allows for the combining of the camera probe for measuring the visual quality of semolina and flour color (L*, a*, and b*) in the CIE color space and the detection of black and brown specks. System flexibility facilitates the combining of different products and probe positions.

The unit can detect even a small change in color and contamination of the product due to leakages, such as ruptures in the sifter or purifier sieve. An early detection in this scenario is very helpful. The software offers current readings in addition to the trending charts. The unit needs calibration only once for color measurement with a reference method. It has the flexibility of being integrated as part of the process control system or could be used as a stand-alone. Residual starch content in bran can also be measured if so desired as an option. Changes in starch content would be indicative of yield fluctuations.

When connected to a comprehensive plant automation system, the full potential of online quality monitoring system is further realized in the optimization of production process control enhancing semolina yield while maintaining the desired quality in semolina consistently. Consistency in quality is key to millers and end-users alike, as these processes, for the most part, are automated. Inconsistencies in quality, therefore, end up impacting downstream processing and product quality.

Since there are no improving agents or additives that are added to compensate for quality shortcomings in pasta making, it is therefore very important to deliver the required quality consistently through reliable processing.

## 8. Innovations in Plant Operation, Monitoring, Control, and Digitalization

### 8.1. Plant Operation Using Programable Logic Controllers (PLCs) with Computer Interface

Going back 35–40 years ago, when operating a milling plant, process monitoring and performing control by replacing hardwired electro-mechanical relays with programable logic controllers (PLCs) and with computer interface (the aid of PCs) was considered a major advancement in plant automation. During this period, some plants were being touted as “lights out” plants, where no personnel were present during the night shift. Although such possibilities were very advanced for the time, the system was only able to shut the equipment down in a failsafe manner, at best, and the status was communicated to the operator by alarm.

The use of digitalization with artificial intelligence, machine learning, cloud computing, and internet of things (IoT) has brought about changes in almost every aspect of the industrialized world. Among the most significant advancements in durum milling are innovations in digital technologies and its application in developing a very powerful and comprehensive plant automation system. Plant monitoring in real time provides enhanced productivity with optimized quality assurance. The leading equipment manufacturing and process solution company Bühler Inc. of Switzerland [31] offers such a system, along with comprehensive support in providing guidance to the milling companies in their pursuit of digital transformation.

Digitalization using a holistic approach of integrating business processes such as Enterprise Resource Planning (ERP), maintenance, quality monitoring, yield management system, and others has made it possible to have enhanced capabilities. These capabilities include increased efficiency, transparency, and traceability, along with controlling the entire production in real time from anywhere. The system integrates data in a central data base system that are used for the optimization of the production process on an ongoing basis. In situations where problems are detected, the system facilitates prompt intervention.

The availability of the automation system, along with offerings of digital services by the equipment manufacturers, has emerged as an area of development of great significance.

### 8.2. Self-Adjusting or Smart Mill

The operation of a newly developed milling plant capable of using its own process parameters in a closed loop to optimize its production has been widely reported in trade publications [56,57,58]. The most advanced blend of engineering with the application of digitalization is being harmonized in a highly technically advanced way in the development of the most innovative milling technology of today (Buhler Group) [31]. We are now in a digitalized world, with the application of machine learning and AI enabling us to navigate through complex process optimization. This development of the self-adjusting mill will be the precursor to the smart mill [56].

As was described in the preceding sections, the wheat-milling operation has evolved into a very highly sophisticated technology with the advancements in process and equipment development. Milling companies are expected to generate finished product quality, as specified, consistently in food-safe conditions. The present-day expectations of the above would also require production in a sustainable way. This would mean the use of less energy and the reduction of waste. The use of innovation in advanced automation, sensors’ application, and digitalization would potentially help in improving the reduction of waste and energy use with a certain strategic and novel approach in design development.

In this self-adjusting plant, as reported, the system processes a large volume of data points, exceeding 15,000, covering all aspects of the production process for optimization on an ongoing basis.

#### 8.2.1. Building Design

The modular design of the mill is kept in mind with the “plug and play” concept of the equipment, allowing for the reduction in installation time by 30%. A more innovative building design helped reduce the building volume further by cutting the building cost by an additional 30%. The application of an energy-efficient fully integrated grinding system with the resource of full digitalization further helped in the reduction of energy costs, as targeted. The mill is designed for optimum performance, while also allowing for easy accessibility of the equipment for maintenance. The pneumatic conveying of products throughout the plant is carried out by blower units which are preassembled

#### 8.2.2. Consistent Process Optimization

The key parameters of the incoming wheat are checked by the sensors as the wheat comes into the mill, and the sensors in the grinding system check it again and recalibrate the settings based on the changing characteristics of the wheat as appropriate. The ability of the process to optimize its setting on its own is the key feature of the self-adjusting mill [56].

#### 8.2.3. Operation of the System

The sensors send the data to an advanced plant automation system that performs the routine process monitoring and control operation. Sensors also send the data to an IoT hub where algorithms are performed, comparing present production and process parameters with the past. This enables the milling plant to perform optimally, thus achieving the most consistent product.

Additional service modules include Temperature and Vibration Management Service (TVM), Yield Management System (YMS), Error and Downtime Analysis (EDA), and Overall Equipment Effectiveness (OEE). These modules feed data on an ongoing basis on machine and process trends, potential maintenance matters, and how machine performance relates to quality and efficiency [56].

#### 8.2.4. Application of Block Chain Technology

With the planned use of blockchain in the future, the customers of the milling company can benefit from accessing/viewing the production parameters in real time as part of the product certification process. This is achieved through a seamless interface from laboratory systems to plant automation system and IoT hub and then to the milling company’s customer through blockchain. The secure data handling of blockchain provides the transparency desired by customers, as they can verify the production parameters used for the production of their product. This would likely result in the reduction and simplification of product sampling and testing. The end result is that this process, while simplifying product testing, will enable a consistent, retraceable food-safe product [56].

#### 8.2.5. Future Development

The development reported with the self-adjusting mill has been phenomenal. Despite the achievement, it is still referred to as a precursor to a fully developed “Smart mill” of the future. Based on the reports, there is a need to further understand the high volume of data being generated by sensors relating to the process for a better understanding of all the machine parameters required for the manufacturing of high-quality end products.

This appears to be very futuristic; nevertheless, such a milling plant is operational. Although the concept and the tools are currently being applied in a flour milling unit, the technology will be transferrable to a durum milling facility in the future.

## 9. Food Safety

Food safety is a very comprehensive subject with a large volume of documented procedures that continue to evolve in step with developments and changing environment. Hufford (2018) [59] stated that it is a living and breathing document [59]. The reference to food safety here is made within the context of how recent technological advances in durum wheat milling are helping to address food safety issues.

The downstream processing of durum wheat semolina helps in eliminating or reducing the risk of any microbiological contamination that may be present. As most pasta is processed by using an HT (high-temperature) drying process, there is little risk of contamination in the pasta [28].

Since the main source of exposure is through the contamination of incoming grain with soil, dirt, and plant material, as well as the presence of damaged and diseased kernels, molds, fungi, surface contaminants and infested kernels, a thorough inspection of the incoming wheat is an essential part of the routine before the wheat gets precleaned and sent to bins for safe storage. If the wheat received shortly after the harvest has an elevated moisture level that is unsafe for storage, a proper aeration system may be necessary to dry it down to the moisture level that is safe for storage.

Out of the three typical hazards identified [59]—biological, chemical, and physical—for wheat milling plants, all of them are related to wheat to a greater or lesser extent.

The biological hazards of durum wheat could include the cadmium level; contamination with mycotoxins, such as DON and ochratoxin (OTA); and *E. coli* and Salmonella contamination [60].

Chemical hazards include the overtreatment of crop with fungicide, pesticides, and insecticides to control weeds, mold, pests, and insects.

Physical hazards include all types of foreign material, such as chunks of wood, stones, metals, glass, and rubble, which, if they remain present in the wheat stream, they may break down or disintegrate in smaller pieces and pose a serious safety hazard.

In the description of wheat preparation and milling equipment, the design and function with reference to food safety has been adequately covered. Additionally, it is worth noting that magnets are installed at various critical points, especially before any cleaning machine that uses friction as a principle for its operation, such as scourers and pearlers. It has also been noted that pearlers significantly lower the microbial load before wheat is milled and contamination extends to milled products. A study noted that a substantial reduction of bacterial and mold load from >1 log to >5 log was achieved by treating tempering water with antimicrobial agents [61]

A big part of food safety procedures involves the documentation and maintenance of records and traceability. In the present environment of automation and digitalization, this part of the food safety function is easily performed.

The demand for food safety has always been growing for a long time, becoming increasingly important, since cases of foodborne illnesses began being linked to wheat flour. Following the reporting of the detection of pathogens in flour [62] and several reports of a similar nature, there was a raised heightened level of awareness on the issue of food safety. Raw wheat flour may be exposed to Salmonella or Pathogenic *Escherichia coli* (*E. coli*); therefore, raw flour, dough, or batter should not be eaten or tasted [60,63,64].

Food safety has become an important part of quality assurance and protection. The associated functions of documenting, maintaining a required paper-trail, and preparation for audits are time-consuming and a drain on resources, and there are operating expenses when carried out manually. Since production-process- and quality-related data are recorded in the central database, they are all available and traceable, as required, enabling the preservation of related proof-of-product traceability and quality information.

Such a possibility has never been conceived of before. The recent developments have been primarily concentrated in this area of digitalization of all the food processes, enabling the operations’ savings on energy and resources while furnishing valuable production data.

## 10. Recent Trends in the Milling Industry

### 10.1. Health and Nutrition

The trends related to low-carb, gluten-free, high-fiber, whole-grain, organic, and enriched pasta with a variety of nutritional components have been in the market for some time, and such products have a secure shelf space. While the pasta produced from 100% durum semolina is affected in terms of market share because of gluten-free and other varieties of pasta, it does provide an opportunity to durum millers in diversifying their production involving these other components

### 10.2. Environmental Sustainability

- Major milling-equipment manufacturers have taken steps to follow sustainable practices; for example, Bühler Inc. set up their corporate target of cutting water, energy, and food waste by 50% in their customer-value chains by 2025 [56]. Ocrim, a major milling equipment manufacturer based in Italy is using an intelligent energy-saving system with high-efficiency motors and other measures to build energy-efficient mills [65].
- Barilla, the largest pasta maker, requires wheat produced under their established farming code to ensure that quality, economic, environmental, and social sustainability are met [66]. Durum millers have established their own goals and have aligned their sustainable practices accordingly by cutting back on energy, using renewable energy [67], and reducing waste. Millers are also partnering with the farm community in support of farm practices that can improve the environment through regenerative agriculture.

### 10.3. Larger Production Units

There is a growing trend of construction of larger-capacity milling units. Milling companies, particularly those located in a highly competitive environment such as in North America, are leveraging on economies of scale to improve their margins by concentrating their production in large units. Wheat milling, whether common wheat or durum wheat milling, is a low-margin business. The scale of economy has allowed us to take advantage of rationalizing the managing of production, marketing, distribution, and related administrative costs. There is benefit in combining production capacities through mergers [68] to reduce operating costs and improve margins.

## 11. Conclusions

Advances in quality improvement and milling innovation have led to improvements in the functional attributes, food safety, and sustainable production of durum wheat products. General developments in regard to processing equipment and digital application with IoT platforms have transformed the processing ecosystems, radically enabling greater efficiency and the saving of resources. Quick fault detection and timely intervention help in saving production time, cutting down on interruptions, and eliminating wastage, all of which contribute to more efficient and sustainable production. Sustainability has also been enhanced by efforts toward process simplification and improving building design, which have helped to reduce energy needs. Finally, the application of digitalization has created benefits of consistency in production, quality, transparency, documentation, and traceability, and this has supported the efforts of processors to improve their productivity, ensure food safety, and enhance sustainability efforts.

## Figures and Tables

**Table 1:** Selected quality parameters of Canadian durum export aggregates.

Parameters	2006–2010	2011–2015	2016–2020
Total Yellow Pigment Content (semolina), ppm	7.5–9.0	8.5–10	9.0–11
Gluten Index (Semolina), %	40–55	60–85	65–85
Cadmium (Grain), ppb	140–80	85–65	85–65

Source: www.grainscanada.gc.ca (accessed on 1 February 2022).

**Table 2:** Tolerances of selected grading factors for CWAD wheat.

Grading Factors	No. 1	No. 2	No. 3	No. 4
Test Weight, kg/hL	80	79	78	75
HVK, %	80	60	40	No Minimum
Ergot, %	0.02	0.02	0.04	0.04
Fusarium Damage, %	0.5	0.5	2.0	2.0

Source: www.grainscanada.gc.ca (accessed on 1 February 2022).

**Table 3:** Comparative development of cleaning equipment of significance.

Sequence of Removal of Impurities and Related Advantage	Traditional Cleaning System	Contemporary Cleaning System	Advantages
Larger and finer impurities than wheat are removed first. Bulk of the impurities is removed here, reducing the load on subsequent equipment	Grain separator, using screens for separation	Combi-Cleaner/Vitaris -combines all 4 functions in one	Both the Combi-Cleaner and Vitaris perform 4 functions in a single machine, helping in reduced space requirements, energy savings, and supervision. Vitaris provides additional feature of modularity, allowing for the selection of any desired combination of these 4 functions as per the needs of the plant and improved energy efficiency
Lighter impurities than wheat are removed here, improving the cleaning efficiencies of the subsequent equipment	Aspiration channel with air-recycling		
Separation of heavy and mixed wheat streams. Heavy stream requires removal of stones and no further cleaning; only a smaller fraction of mixed stream with lighter seeds require cleaning	Concentrator, using air and screens for separation		
Stones, glass, and metals of similar size as wheat that could not be removed by screens are removed here. Early removal protects subsequent equipment damage and helps in strict adherence to food safety	Destoner, using density as the basis of separation		
Longer seeds than wheat—oats, barley, and shorter seeds such as cockle and wild buckwheat—are removed here. Since most of the impurities are removed already, as mentioned above, this allows precise adjustment to be made here for the removal of these materials	Indented cylinders, using shape as the basis of separation	Optical sorters, using optical measurements, color, shape, and size	Optical sorters help reduce the percentage of screenings due to efficient and effective removal of the rejects with no loss of wheat. This helps increase the yield of cleaned wheat; it is also space saving and energy saving
Heavier and lighter fraction separation/removal of ergot, oats, and barley	Gravity tables/ table separators		
Surface contaminants	Scourers, using friction as the basis of cleaning	Pearlers, using abrasion/friction	Removes significant percentage of bran, surface contaminants, microbial load, and superficial discoloration

**Table 4:** Milling passages for coarse and fine semolina production.

Semolina Granulation	Break Passages	Grading	Purification	Sizing	Reduction	Conversion
Coarse semolina	B1–B7F	Div 1–Div 4	S1–S26	D1–D7	RED 1–RED 3	
Fine semolina	B1–B5	Div 1–Div 2	S1–S12	D1–D3	RED 1–RED 2	C1–C4

**Table 5:** Milling surface allocation of durum semolina mills for fine and coarse semolina ^1^.

	Fine Semolina	Coarse Semolina
Granulation, μm	355–0	630–125
Roll, mm/100 kg/24 h	11.3–12.5	15–18
Purifier, mm/100 kg/24 h	3.7–4.5	5–7
Sifter, m^2^/100 kg/24 h	0.060–0.064	0.062–0.068

^1^ Based on expected average commercial data.

**Table 6:** Semolina ash content regulation.

	Max	Max (Dry Moisture Basis)
Country	Moisture, %	Ash, %
Italy (semola) ^1^	14.5	0.90
France (SSSE) ^2^	14.5	0.80
USA ^3^	15.0	0.92

^1^ PRESIDENTIAL DECREE N° 187, dated 9 February 2001 (Official Journal n. 117, of 22 May 2001). Article 2. Durum wheat milling products. More details provided in Article 2 [46]. ^2^ Fixing the characteristics of durum wheat semolina and pasta as modified by the by-laws of 22 July 1959, 13 August 1974, and 6 December 1974. More details provided in Article 3 Superior durum wheat semolina SSSE UNAFPA July 2001 [47]. ^3^ Code of Federal Regulation. Title 21. Sec. 137.320 Semolina. Food and drugs. [48].

**Table 7:** Advances in three principal pieces of milling equipment used in a durum milling operation.

Modern Equipment	Features	Advantages
Roller Mills	Advanced use of sensors and automation. Improved design with high food safety standards and energy efficient drives. Quick installation and space saving. Roller mills supplied with a touch screen panel for operational control which can also be remotely accessed using a plant computer or through a wireless connection with a tablet or mobile.	Stable and uniform control on grinding. Higher sanitary standards and improved food safety. Improved standards of sustainable operation with energy- and space-saving features. Ease of operation and accessibility.
Plansifters	large sieving area with optimized space utilization. Top sanitation and Hygiene. Sturdy construction with energy efficient operation. Stable design with lightweight energy efficient motor.	Increased sifting capacity due to increased sifting area Improved food safety. Low maintenance. Sustainable operation with lower energy requirement and improved sifting capacity
Purifiers	High specific capacity at same space. High sanitary standard completely enclosed. Energy efficient.	Improved semolina purification. Improved food safety design. Increased output. Sustainable operation with energy efficiency and more output on smaller footprint.

**Table 8:** Quality attributes measured online.

Property	Durum Wheat	Semolina and Flour
Moisture	✓	✓
Protein/Gluten	✓	✓
Ash	✓	✓
Color L*, a*, b*		✓
Specks Black and Brown		✓
Particle size distribution		✓

**Table 9:** Specifications related to particle size distribution.

Semolina Type	European Semolina (Special)	Common Semolina	Handmade Couscous	Industrial Pasta and Couscous	Extra Fine Semolina Special Bread
	µm	µm	µm	µm	µm
Granulation	630–200	425–125	1000–600	400–212	300–160

## Data Availability

The data presented in this study are available on request from the corresponding author.
